# Supplementary material for: Gram-Positive Bacteria-Like DNA Binding Machineries Involved in Replication Initiation and Termination Mechanisms of Mimivirus
Source: Viruses. 2019 Mar 17;11(3):267. doi: 10.3390/v11030267 (PMC6466248; doi:10.3390/v11030267)
Supplement: Supplementary file 1 [file viruses-11-00267-s001.zip › Supplementary_Materials.pdf]

viruses

MDPI

Supplementary Materials for:

Gram-positive bacteria-like DNA binding machineries involved in the replication initiation and termination mechanisms of Mimivirus

Motohiro Akashi* and Masaharu Takemura

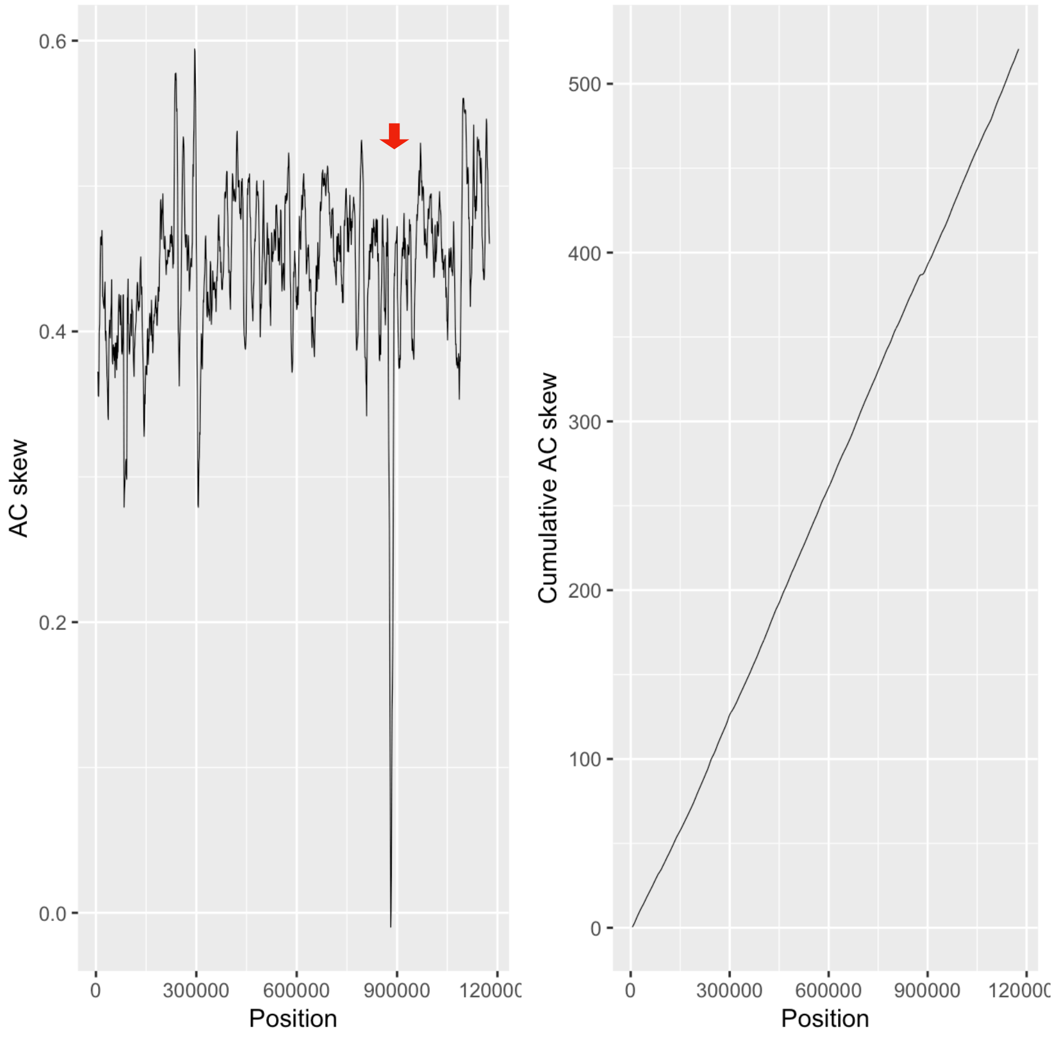

Figure S1. AC skew analyses of the *Acanthamoeba polyphaga mimivirus* (APMV) genome. AC skew plot (left) and cumulative AC skew plot (right) of APMV genome (AY653733.1). Red arrows indicate the lowest peak (881,000 ± 5,000 bp), which corresponds to one of the lowest values on the GC skew plot (882,000 bp).

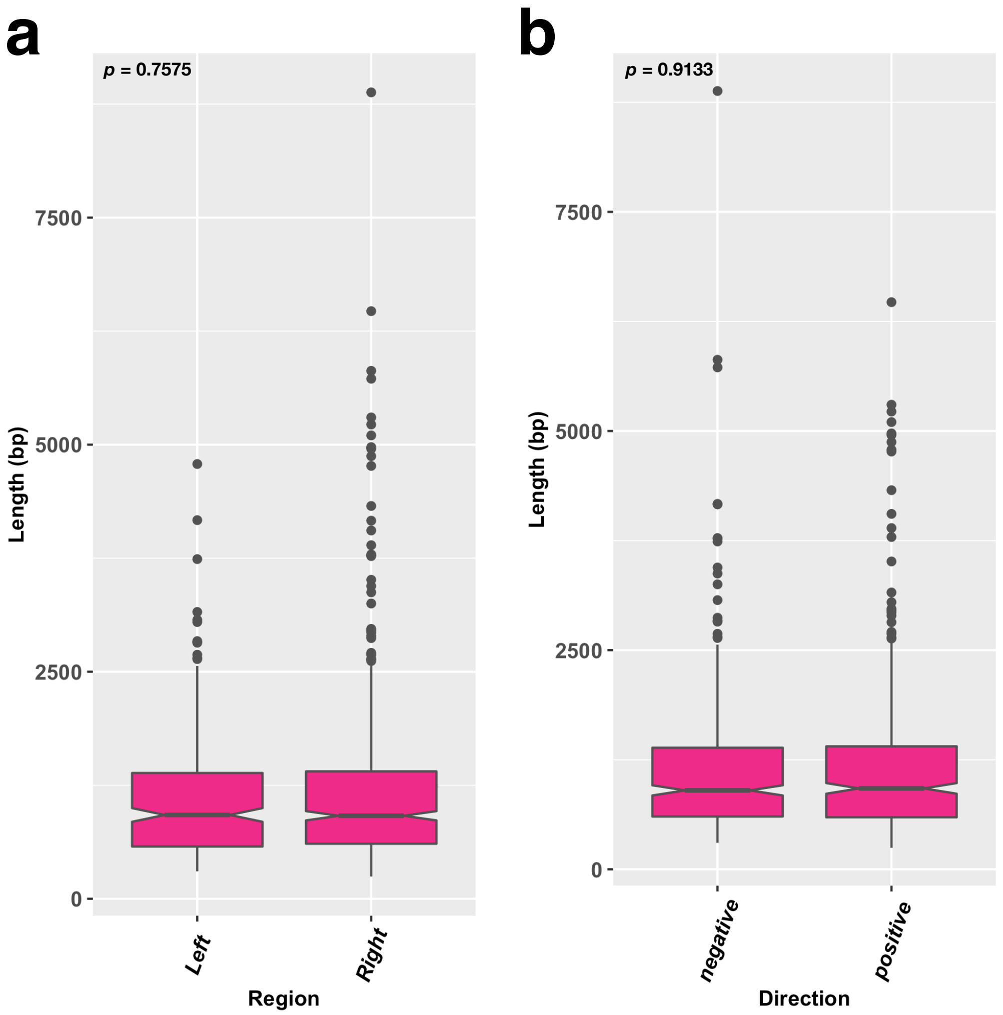

Figure S2. Correlation of the coding sequence (CDS) lengths of the left and right sides from the estimated *ori*-region. (**a**) CDS lengths of the left and right sides from the estimated origin (380,698 bp). (**b**) CDS lengths of the positive and negative direction in the APMV genome (AY653733.1)). The *p*-values on the top left of each graph indicate the statistical differences between each of the two group-based results of a KS test.

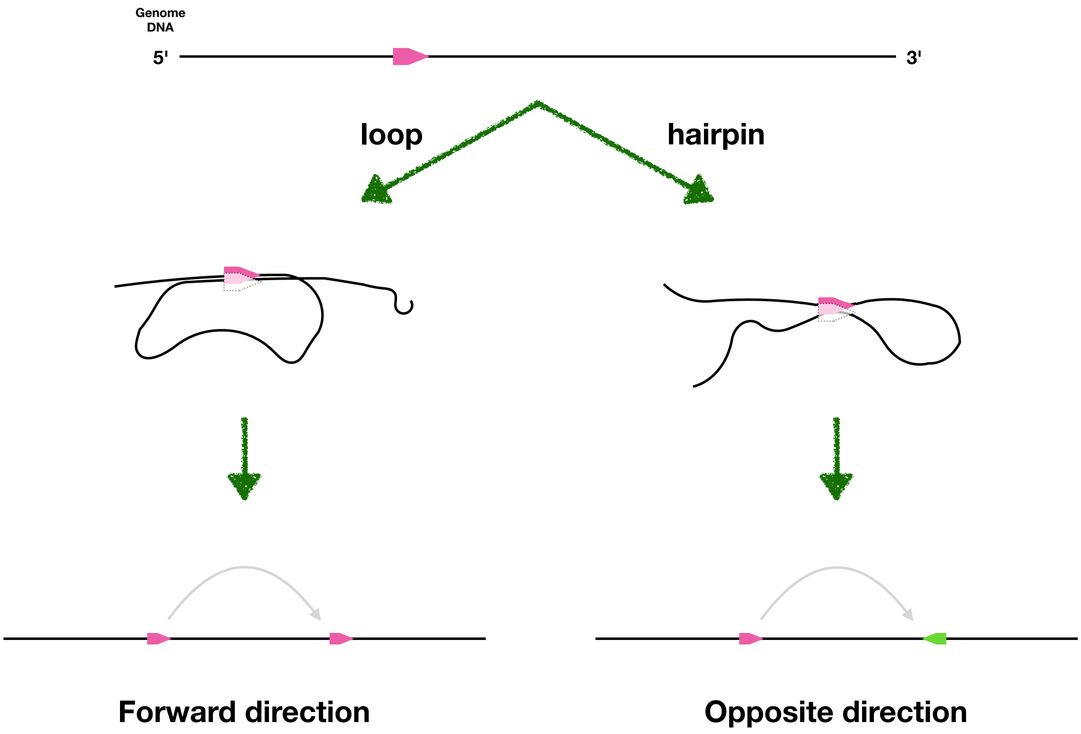

Figure S3. Models of the generation of paralogous genes far from each other and their directions on the linear genome. Pink/green arrow indicate paralogous genes with forward/opposite direction. A loop (left) or hairpin (right) structure would cause the generation of paralogous genes with forward or opposite directions via homologous recombination.

© 2019 by the authors. Submitted for possible open access publication under the terms and conditions of the Creative Commons Attribution (CC BY) license (http://creativecommons.org/licenses/by/4.0/).
